# Effect of Temperature Changes on Serum Protein Adsorption on Thermoresponsive Cell-Culture Surfaces Monitored by A Quartz Crystal Microbalance with Dissipation

**DOI:** 10.3390/ijms19051516

**Published:** 2018-05-18

**Authors:** Jun Kobayashi, Yoshinori Arisaka, Nobuhiko Yui, Yoshikatsu Akiyama, Masayuki Yamato, Teruo Okano

**Affiliations:** 1 Institute of Advanced Biomedical Engineering and Science, Tokyo Women’s Medical University (TWIns), 8-1 Kawadacho, Shinjuku-ku, Tokyo 162-8666, Japan; akiyama.yoshikatsu@twmu.ac.jp (Y.A.); yamato.masayuki@twmu.ac.jp (M.Y.); tokano@twmu.ac.jp (T.O.); 2 Department of Organic Biomaterials, Institute of Biomaterials and Bioengineering, Tokyo Medical and Dental University, 2-3-10 Kanda-Surugadai, Chiyoda-ku, Tokyo 101-0062, Japan; arisaka.org@tmd.ac.jp (Y.A.); yuiorg@tmd.ac.jp (N.Y.); 3 Cell Sheet Tissue Engineering Center and Department of Pharmaceutics and Pharmaceutical Chemistry, University of Utah, 30 South 2000 East, Salt Lake City, UT 84112, USA

**Keywords:** thermoresponsive polymer, poly(*N*-isopropylacrylamide), quartz crystal microbalance, thermoresponsive cell culture surface, cell sheet

## Abstract

Thermoresponsive cell-culture polystyrene (PS) surfaces that are grafted with poly(*N*-isopropylacrylamide) (PIPAAm) facilitate the cultivation of cells at 37 °C and the detachment of cultured cells as a sheet with an underlying extracellular matrix (ECM) by reducing the temperature. However, the ECM and cell detachment mechanisms are still unclear because the detachment of cells from thermoresponsive surfaces is governed by complex interactions among the cells/ECM/surface. To explore the dynamic behavior of serum protein adsorption/desorption, thermoresponsive surfaces that correspond to thermoresponsive tissue-culture PS dishes were formed on sensor chips for quartz crystal microbalance with dissipation (QCM-D) measurements. X-ray photoelectron spectroscopy (XPS) measurements and temperature-dependent frequency and dissipation shifts, Δ*f* and Δ*D*, using QCM-D revealed that the thermoresponsive polymers were successfully grafted onto oxidized, thin PS films on the surfaces of the sensor chips. Increased amounts of adsorbed bovine serum albumin (BSA) and fibronectin (FN) were observed on the thermoresponsive polymer-grafted surfaces at 37 °C when compared with those at 20 °C because of enhanced hydrophobic interactions with the hydrophobic, thermoresponsive surface. While the calculated masses of adsorbed BSA and FN using QCM-D were 3–5 times more than those that were obtained from radiolabeling, the values were utilized for relative comparisons among the same substrate. More importantly, the thermoresponsive, dynamic behavior of serum protein adsorption/desorption was monitored using the QCM-D technique. Observations of this dynamic behavior revealed that the BSA and FN that were adsorbed at 37 °C remained on both surfaces after decreasing the temperature to 20 °C.

## 1. Introduction

Physisorption of biomolecules and cells onto the surfaces of biomaterials occurs irreversibly through nonspecific interactions. Our laboratory covalently introduced a thermoresponsive polymer, poly(*N*-isopropylacrylamide) (PIPAAm), onto surfaces to regulate the interactions with biomolecules and cells by temperature changes [1,2]. PIPAAm exhibits reversible soluble/insoluble changes in an aqueous milieu at the phase-transition temperature of 32 °C [3]. Tissue-culture polystyrene (PS) surfaces that were grafted with PIPAAm by electron-beam irradiation enable attached cells to be reversibly detached [4,5]. Based on this technology, a thermoresponsive cell-culture dish has been produced and is commercially available as UpCell^®^. At 37 °C, cells were grown to confluence on hydrophobic surfaces that were covered by dehydrated PIPAAm-grafted surfaces. When the temperature was lowered to 20 °C, the surfaces became hydrophilic, according to hydration of the grafted PIPAAm, and a confluently cultured cell monolayer was detached as a single cell sheet. The detached cell sheet holds cell-cell junctions and the extracellular matrix (ECM) beneath the sheet [6] and it can be easily transplanted by attaching it to targeted tissue surfaces and/or another cell sheet. Layered cell sheets form three-dimensional tissues [7] and are transplantable to treat damaged tissues and organs [8,9,10,11,12,13]. 

Low temperature-induced detachment of adhered cells from a PIPAAm-grafted surface requires physicochemical changes on the surface and biochemical processes within cells. The addition of reagents to suppress cellular metabolism, signal transduction, and cytoskeletal activities in a culture medium inhibits the detachment of cells. The PIPAAm-grafted surface exhibits hydrophilic property at 20 °C [14,15]; thus, surface properties change from hydrophobic (37 °C) to hydrophilic (20 °C) in order to trigger the detachment of the adhered cells. Subsequently, pulling forces are generated by the dynamic motion of the cytoskeletons within cell sheets, resulting in detached cell sheets shrinking relative to the original size. During the detachment of cells, ECMs, such as fibronectin (FN), are released from the surface and they are co-located with the detached cells [6,16]. Recently, we developed a heparin-immobilized thermoresponsive cell-culture surface to stimulate cellular receptors and the non-enzymatic recovery of cultured cells as a contiguous sheet [17,18]. Immobilized heparin molecules on shrunken PIPAAm can capture heparin-binding growth factors. We found that both the ECMs and heparin-binding growth factors detached from the surface upon lowering the temperature to 20 °C. This indicated that the affinity binding between immobilized heparin and the growth factors was ruptured without any chemical cleavage. Thus, the detachment of cells from the thermoresponsive surfaces was governed by complex interactions among the cells/ECMs/surfaces. 

The final goal of this work is to develop a methodology to explore the dynamic process of cell and ECM detachment from thermoresponsive surfaces. The dynamic process of cell detachment has been investigated by observing the kinetics of cell detachment [19,20,21]. In contrast, there have been no reports on the monitoring of ECM adhesion and de-adhesion during cell detachment processes. The utilization of sensing technologies, such as a quartz crystal microbalance with dissipation (QCM-D) [22,23,24], surface plasmon resonance (SPR) [25,26], and reflectometric interference spectroscopy (RIfS) [27] facilitates monitoring the temperature-dependent dynamics of PIPAAm-grafted surfaces on inorganic substrates, such as Au and quartz. However, the same surface composition as that of PS-based thermoresponsive surfaces is required to fully understand the ECM and the cell detachment mechanisms. The properties of the basal substrate under the thermoresponsive polymer layers have a large influence on the temperature responsiveness [28], because thin, surface-grafted thermoresponsive polymers interact with the basal substrate and change the dehydrated aggregation force at 37 °C. Thus, in this study, PIPAAm- and heparin-immobilized thermoresponsive surfaces were formed on sensor chips for QCM-D to replicate thermoresponsive tissue-culture PS dishes and were utilized to explore the thermoresponsive dynamic behavior of serum protein adsorption/desorption. Here, FN and bovine serum albumin (BSA) were selected as model serum proteins [29]. Typically, fetal bovine serum (FBS) is supplemented in the cell culture medium for maintaining the adhesion and the survival of cultured cells. FN is a serum protein that mediates the attachment of cells through the binding between cell-binding domains of adsorbed FN and integrin receptors on the cells. By contrast, BSA is the most abundant serum protein, and adsorbed BSA on the surface of biomaterials reduces the cell attachment because of the lack of cell-binding domains. Therefore, it is expected that the development of a methodology to monitor the adsorption/desorption behavior of both FN and BSA on the thermoresponsive surfaces leads to revealing the ECM and cell detachment mechanisms.

## 2. Results

### 2.1. Preparation of Thermoresponsive Polymer-Grafted PS (Polystyrene) Surfaces on Sensor Chips

**Figure 1:**
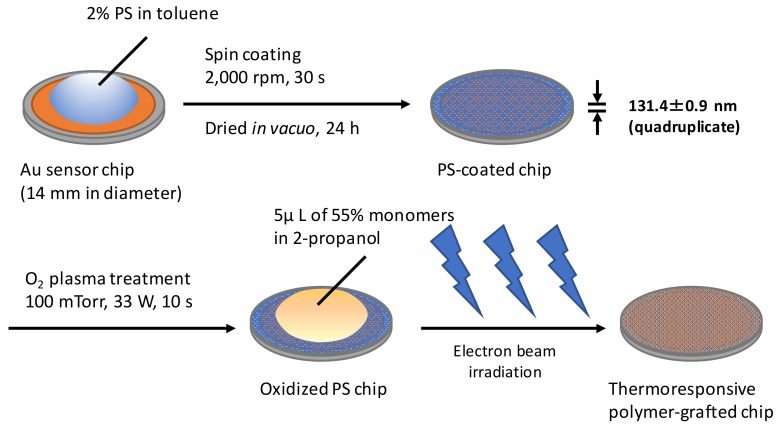
Schematics of the preparation of polystyrene (PS)-coated sensor chips and their surface modification with thermoresponsive polymers by electron-beam irradiation.

Thermoresponsive polymer-grafted PS surfaces on sensor chips were prepared by spin coating PS and covalently grafting the thermoresponsive polymer by electron-beam irradiation (Figure 1). At first, a PS layer with an optimal thickness was formed on the Au sensor chips. The glass-transition temperature of a thin PS film, i.e., thickness below 50 nm, is thickness-dependent [30]. In addition, the surface viscoelastic property and the surface dynamic storage modulus on the PS film increased with the decreasing thickness below 80 nm [31]. Therefore, a PS film with a thickness of more than 100 nm resembles the bulk in terms of both the glass transition temperature and surface viscoelastic properties. Spin coating using a 2% solution in toluene with 2000 rpm formed a thin PS film with a thickness of 131.4 ± 0.9 nm, and the thickness was determined using spectral interferometry with quadruplicate measurements. The thickness of the spin-coated PS film was comparable to that in a previous report [32].

**Figure 2:**
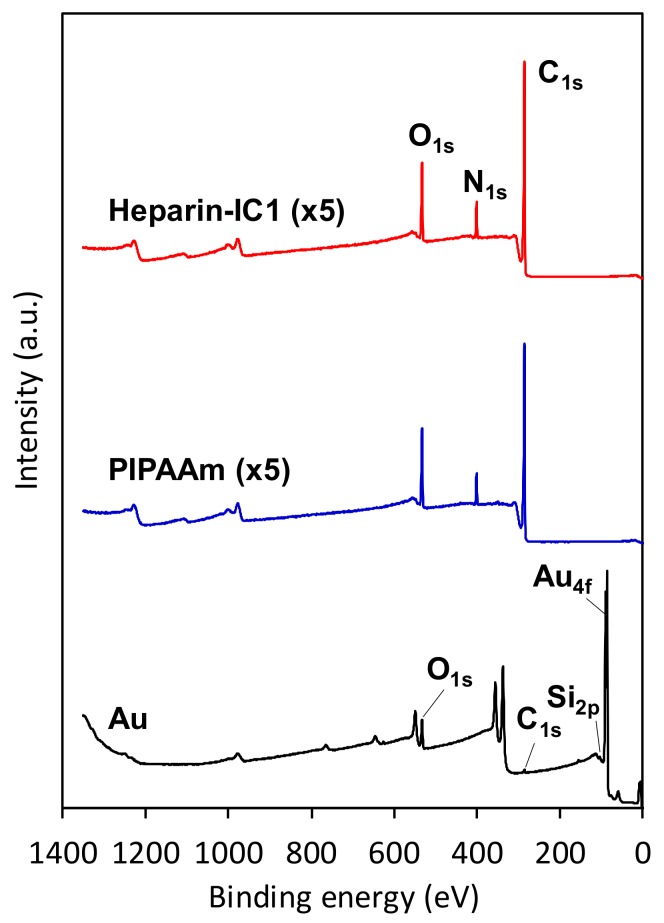
X-ray photoelectron spectroscopy (XPS) survey spectra of thermoresponsive polymer-grafted PS (poly(*N*-isopropylacrylamide) (PIPAAm) and Heparin-IC1) and bare Au surfaces on sensor chips. Take-off angle, 90°.

The introduction of thermoresponsive polymers onto sensor chip surfaces was confirmed by surface elemental analyses using X-ray photoelectron spectroscopy (XPS). The XPS survey spectra of PIPAAm, heparin-immobilized poly(IPAAm-*co*-CIPAAm) (Heparin-IC1), and bare Au surfaces are shown in Figure 2. Peaks for C, N, and O were found on the PIPAAm and Heparin-IC1 surfaces. No signals that were derived from the basal sensor chips were detected for either surface, indicating that the surfaces were uniformly covered with PS and/or the thermoresponsive polymer. The detected surface compositions of elements C, N, O, S, and Au are summarized in Table 1. The N/C ratios that were calculated from the XPS data for the PIPAAm and Heparin-IC1 surfaces were not absolutely identical to the theoretical values. However, a higher amount of nitrogen and a lower amount of oxygen were observed on the polymer-grafted surfaces when compared with those on the oxidized PS surface. Therefore, the PIPAAm and Heparin-IC1 surfaces were considered to be covered with a very thin polymeric layer. Although the detected sulfur contents on the three surfaces were small, a slightly higher amount of sulfur was observed on the Heparin-IC surface when compared with that of the other two surfaces. This increase in sulfur implied the incorporation of the sulfuric acid groups derived from heparin. These data proved that the thermoresponsive polymer and heparin were successfully grafted onto the surfaces of the sensor chips.

### 2.2. Temperature-Dependent Changes in Δf and ΔD Values on PIPAAm-Grafted Surfaces

**Figure 3:**
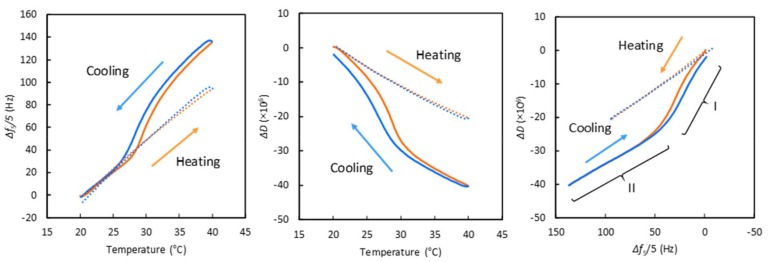
Temperature-dependent changes in Δ*f* (**left**) and Δ*D* (**middle**) and Δ*D* vs. Δ*f* plots (**right**) for the fifth overtone in PBS on bare Au (dash) and PIPAAm surfaces (solid) during a heating-cooling cycle. Heating and cooling rate, 0.1 °C/min. Flow rate, 50 µL/min.

To check the temperature dependency during QCM-D measurements, we monitored the temperature-dependent frequency and dissipation shifts, Δ*f* and Δ*D*, respectively, of PBS on PIPAAm and bare Au surfaces. Figure 3 shows the temperature-dependent changes in Δ*f* and Δ*D* on the bare Au and PIPAAm surfaces for a heating-cooling cycle at 0.1 °C/min, and the PIPAAm-grafted surface was nearly quasi-static [33]. On the Au surface, the Δ*f* values linearly increased with increasing temperature, whereas the Δ*D* values linearly decreased. This trend was in agreement with that in a previous report [22]. Increasing Δ*f* and decreasing Δ*D* values are due to the decreasing viscosity and water density with the increasing temperature [22,34]. There was no hysteresis between the heating and the cooling processes on the Au surface. In contrast, the plots of Δ*f* and Δ*D* vs. temperature on the PIPAAm surface exhibited inflection points at approximately 29 °C (heating) and 26–28 °C (cooling), while linear shifts were observed on the Au surface. These changes in Δ*f* and Δ*D* around the inflection points were considered to be due to hydration/dehydration alteration of the grafted PIPAAm chains through a temperature-induced phase transition.

In Figure 3, right, the Δ*D*−Δ*f* plots for the PIPAAm surface are divided into two regions: steeper slope (region I) and same slope as the Au surface (region II). Generally, higher dissipation per frequency values indicate greater changes in rigidity and viscoelastic properties [35]. The slopes on the Au surface were attributed to temperature-dependent changes in the viscosity and the density of water, not surface rigidity and viscoelastic changes. In contrast, the steeper slopes in region I were due to changes in rigidity and viscoelasticity on the PIPAAm surface, which indicated the swelling/deswelling alteration of grafted PIPAAm chains below the phase-transition temperature. On the other hand, no obvious changes in the properties of the PIPAAm surface were observed in region II because the slope was the same as that on the Au surface. This implied that the dehydrated PIPAAm surface exhibited no changes above the phase-transition temperature.

### 2.3. Estimation of Adsorbed Serum Proteins by QCM-D Measurements Using the Voigt Model and Sauerbrey Equation

**Figure 4:**
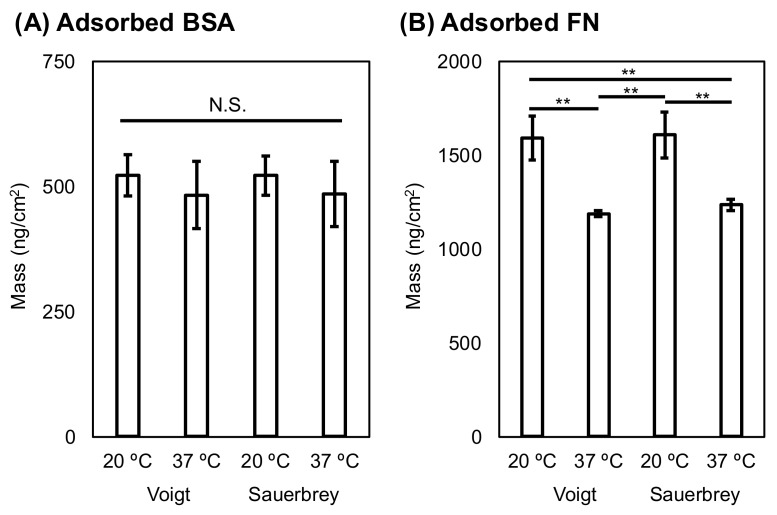
Estimation of adsorbed bovine serum albumin (BSA) (**A**) and fibronectin (FN) (**B**) on oxidized PS surfaces at 20 and 37 °C by QCM-D measurements using the Voigt model and Sauerbrey equation. The bars represent the mean ± S.D. of triplicate experiments. N.S., Not statistically significant, ** Statistically significant, *p* < 0.01.

The estimated thickness values for the adsorbed BSA and FN on the oxidized PS surfaces were calculated using the Voigt model [36] and they are summarized in Table 2. Larger mean values for the thickness of adsorbed BSA and FN were found at 20 °C than at 37 °C. In particular, the thickness of adsorbed FN at 20 °C was larger (*p* < 0.01) than that at 37 °C. In addition, the areal mass (ng/cm^2^) values that were obtained for adsorbed BSA and FN with the Voigt model and the Sauerbrey equation are compared in Figure 4. For adsorbed BSA, there was no significant difference, regardless of the temperature between the Voigt model and Sauerbrey equation. The amounts of adsorbed FN estimated using the different methods were not significantly different for the two groups at the same temperature. These results indicated no difference between the Voigt model and Sauerbrey estimated areal mass values. However, the mass of adsorbed proteins that was measured by the QCM-D technique is overestimated, regardless of the modeling equation because water molecule coupling (e.g., hydration) results in additional mass. Therefore, estimations using the Voigt model and Sauerbrey equation are applicable for calculating the relative areal mass.

### 2.4. Effect of Temperature on the Adsorption of Serum Proteins on Thermoresponsive Polymer-Grafted Surfaces

**Figure 5:**
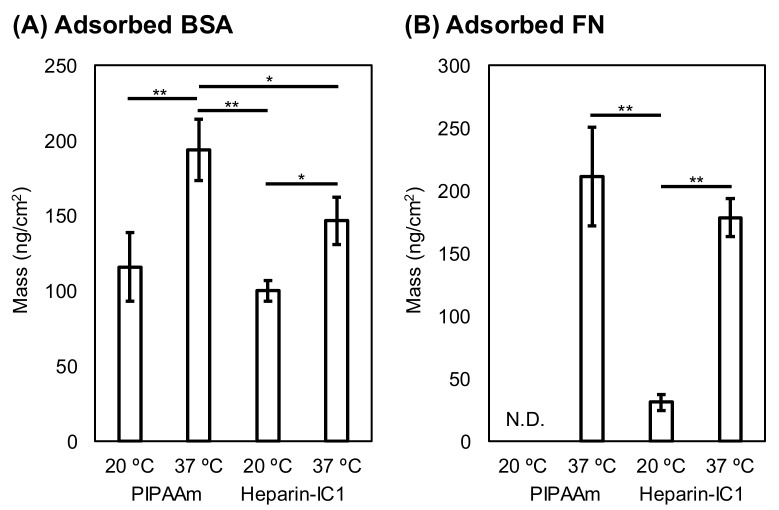
Effect of temperature on the adsorption of BSA (**A**) and FN (**B**) onto PIPAAm and Heparn-IC1 surfaces. The amounts of the adsorbed proteins were estimated by quartz crystal microbalance with dissipation (QCM-D) measurements using the Sauerbrey equation. The bars represent the mean ± S.D. of triplicate experiments. N.D., Not determined. * Statistically significant, *p* < 0.05, ** Statistically significant, *p* < 0.01.

The amounts of adsorbed BSA and FN on thermoresponsive polymer-grafted surfaces were quantified using QCM-D measurements and the Sauerbrey equation. Figure 5 shows the BSA and the FN adsorbed onto PIPAAm and Heparn-IC1 surfaces at different temperatures, 20 and 37 °C. The amounts of adsorbed BSA and FN decreased on both of the surfaces when compared with those on oxidized PS (Figure 4 and Figure 5). Typically, more BSA and FN adsorbed on both thermoresponsive surfaces at 37 °C than at 20 °C. In particular, the difference in the FN adsorption between 20 and 37 °C was significant. This increase in adsorption was mainly due to enhanced hydrophobic interactions with dehydrated thermoresponsive polymers on the surfaces. Negligible adsorption of FN on the PIPAAm surfaces was found at 20 °C, whereas small amounts of FN were adsorbed on Heparin-IC1 surfaces. This was probably due to the affinity of FN for immobilized heparin [37].

For BSA adsorption at 37 °C, reduced amounts were found on the Heparin-IC1 surfaces in comparison with those on the PIPAAm surfaces. The amount of BSA on Heparin-IC1 decreased because of the repulsive force between negatively charged BSA and immobilized heparin, which has sulfuric acid groups.

### 2.5. Dynamic Monitoring of Adsorbed Serum Proteins on Thermoresponsive Polymer-Grafted Surfaces during Stepwise Temperature Changes

**Figure 6:**
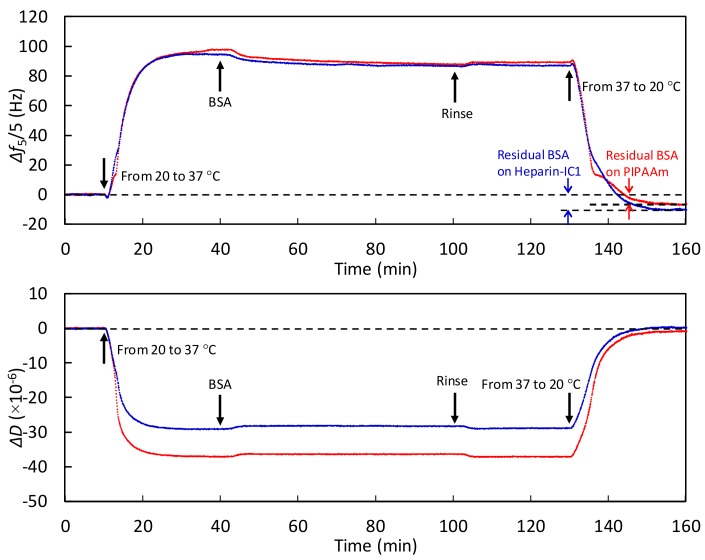
Monitoring temperature-dependent Δ*f* (**upper**) and Δ*D* (**lower**) for the fifth overtone in PBS to analyze the dynamic process of BSA adsorption on PIPAAm (red) and Heparin-IC1 surfaces (blue) during a stepwise temperature change from 20 to 37 °C. Flow rate, 50 µL/min.

**Figure 7:**
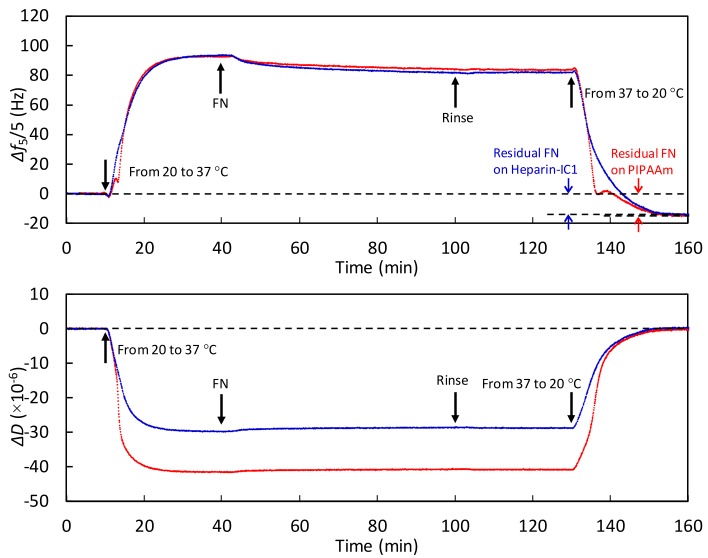
Monitoring temperature-dependent Δ*f* (**upper**) and Δ*D* (**lower**) for the fifth overtone in PBS to analyze the dynamic process of FN adsorption on PIPAAm (red) and Heparin-IC1 surfaces (blue) during a stepwise temperature change from 20 to 37 °C. Flow rate, 50 µL/min.

Figure 6 and Figure 7 show the monitoring of adsorbed BSA and FN at 37 °C and during the stepwise decrease in the temperature to 20 °C on the thermoresponsive polymer-grafted surfaces. Increasing Δ*f* and decreasing Δ*D* values were observed on both surfaces as the temperature was increased from 20 to 37 °C. As shown in Figure 3, left and middle, these changes were mainly due to the decreased viscosity and water density [22], and the loss of surface-bound water through the dehydration of the grafted, thermoresponsive polymers. During the heating process, the degree of change in Δ*f* was almost the same on both surfaces. In contrast, the degree of change in Δ*D* on the PIPAAm surface was greater than that on the Heparin-IC1 surface, which was probably because hydrated heparin molecules contribute to the relative increase in dissipation (i.e., viscoelastically coupled to the solution). The decrease in Δ*f* after the injection of proteins was attributed to protein adsorption on both surfaces.

In addition, the BSA and FN that were adsorbed at 37 °C remained on both surfaces after the temperature decreased to 20 °C. The amount of residual BSA was calculated by the Sauerbrey equation from the difference between the baselines at 20 °C and the plateau after changing the temperature to 20 °C. The residual BSA on the PIPAAm and Heparin-IC1 surfaces was estimated to be 110 and 176 ng/cm^2^, respectively. Similarly, the residual FN on the PIPAAm and Heparin-IC1 surfaces was estimated to be 155 and 194 ng/cm^2^, respectively. Therefore, the QCM-D technique was able to monitor the dynamic process of thermoresponsive protein adsorption.

## 3. Discussion

The objective of this work was to establish thermoresponsive surfaces on sensor chips, which correspond to thermoresponsive tissue-culture PS dishes, in order to explore the thermoresponsive dynamic behavior of serum protein adsorption/desorption. A basal PS film was formed with a thickness of more than 100 nm, and the glass-transition temperature and surface viscoelastic properties of the PS film were similar to those of the bulk [30,31]. Subsequent grafting of thermoresponsive polymers by electron-beam irradiation was carried out, according to the method that was used in previous reports [17,38]. Consequently, thermoresponsive tissue-culture PS surfaces were successfully formed on sensor chips.

There have been a few papers on the effects of temperature change on serum protein adsorption on biomaterial surfaces. Cheng et al. [39] investigated the amount of protein adsorption on PIPAAm-coated poly(ethylene terephthalate) (PET) and bare PET using a radiolabeling procedure. BSA and fibrinogen adsorption on the PET substrate at 37 °C was weaker than that at room temperature. This evidence supports our results in Table 2 and Figure 4, whereas several studies have indicated contrary results, i.e., that heating enhances protein adsorption on inorganic substrates, including silica and silicon [40,41]. The contradictory effect of temperature on protein adsorption is probably due to the differences in the surface chemistry of plastics and silicon oxide, which has polar groups.

The mass of radiolabeled FN that is adsorbed on the surface of tissue-culture PS was reported in previous reports. Grinnell et al. [42] determined that the adsorption of human plasma FN in 22 °C of Tris saline was saturated at 320 ng/cm^2^. Garcia et al. [43] also reported that the saturated value of human FN adsorption in PBS is 350–400 ng/cm^2^. In contrast, the calculated masses of the adsorbed FN in this study using QCM-D were as follows: 1590 ± 116 (Voight) and 1607 ± 122 ng/cm^2^ (Sauerbrey) at 20 °C and 1187 ± 17 (Voight) and 1236 ± 27 ng/cm^2^ (Sauerbrey) at 37 °C. The calculated values in this study are 3–5 times those determined using radiolabeling techniques. Our calculated values are almost the same as those calculated using QCM-D measurements and the Voight model in a previous paper [44]. This evidence indicates that the values that were calculated by QCM-D measurements are overestimated when compared with those calculated by other techniques. This overestimation using QCM-D measurements is due to water coupled with the adsorbed proteins [36]. Therefore, the calculated values in this study are applicable for relative comparisons, not for absolute mass values.

On the thermoresponsive surfaces, PIPAAm and Heparin-IC1, the amounts of BSA and FN adsorption were lower at 20 °C when compared with those at 37 °C, because the hydrated thermoresponsive polymers on the surfaces reduced the adhesion. Surprisingly, FN adsorption on PIPAAm surfaces at 20 °C was negligible, whereas BSA adsorption was observed under the same conditions. In addition, a small amount of FN adsorbed on the Heparin-IC1 surfaces at 20 °C. The degree of adsorption on these surfaces depended on the molecular weight of the proteins. The molecular weights of the proteins that were used in this study are as follows: BSA, 66,300; FN, 440,000 g/mol [41]. Therefore, the larger FN could be inhibited by steric hindrance from the hydrated and swollen thermoresponsive polymer chains.

Finally, we considered why the adsorbed BSA and FN remained on the thermoresponsive polymer-grafted surfaces after reducing the temperature. As the temperature decreased from 37 to 20 °C, the thermoresponsive polymer-grafted surfaces became relatively more hydrophilic than those at 37 °C [4,38]. This alteration induced the detachment of cultured cells from the surfaces. The detached cells and their sheets hold the ECMs, including FN [6,16]. Thus, both the cells and proteins were simultaneously detached by lowering the temperature. However, we previously reported that no release of adsorbed FN without cells from thermoresponsive cell culture surfaces was observed after changing the temperature to room temperature, i.e., below the phase transition temperature, even though 0.5% SDS or 8 M urea solutions were added to treat the surfaces [16]. This evidence is in agreement with the obtained results in Figure 6 and Figure 7. The pulling force that is generated by dynamic cytoskeletal contractions is required for the detachment of FN, which is tightly bound with integrins on the cellular surfaces through affinity based on the dissociation constant between a fibronectin fragment and an integrin α_5_β_1_, i.e., *K*_D_ = 4.4 nM [45].

## 4. Materials and Methods 

### 4.1. Materials

PS petri dishes (Nunc IVF petri dishes) were purchased from Thermo Fisher Scientific (Waltham, MA, USA). *N*-Isopropylacrylamide (IPAAm) was kindly gifted by KJ Chemicals (Tokyo, Japan) and was purified by recrystallization in *n*-hexane. 2-Carboxyisopropylacrylamide (CIPAAm) was synthesized according to a previous report [46]. Sensor chips for the QCM-D (diameter 14 mm, surface area 1.54 cm^2^) were provided by Biolin Scientific (Stockholm, Sweden). 2-Propanol, 1-ethyl-3-(3-dimetylaminopropyl)-carbodiimide hydrochloride (EDC), *N*-hydroxysuccinimide (NHS), and Dulbecco’s phosphate buffered saline (PBS) were obtained from Wako Chemicals (Osaka, Japan) and used as received. 2-Morpholinoethanesulfonic acid monohydrate (MES) was purchased from Dojindo (Kumamoto, Japan). Heparin sodium salt from porcine intestinal mucosa (grade I-A, 180 USP units/mg) and bovine serum albumin (BSA) were obtained from Sigma-Aldrich (St. Louis, MO, USA). The solution of fibronectin (FN) from bovine serum was obtained from Merck (Kenilworth, NJ, USA). To quantify the FN concentration, the absorbance of FN at 280 nm was measured by a NanoDrop 2000 instrument (Thermo Fisher Scientific). The stock concentration of FN was calculated while using an extinct coefficient of 1.28 (g/100 mL)^−1^·cm^−1^ at 280 nm [47,48], and the solution was stored at 4 °C until use. A 4 wt % BSA solution in PBS was prepared as a stock solution.

### 4.2. Preparation of the Thermoresponsive Polymer-Grafted PS Surfaces on Sensor Chips

The surfaces of the sensor chips were cleaned by an oxygen plasma treatment under 13 Pa oxygen with a power of 133 W for 180 s. Two percent of a PS solution in toluene was prepared by dissolving small pieces of a PS dish in toluene and filtering the solution with a PTFE membrane with 0.45 μm pores. A thin PS film (~130 nm) was formed on the cleaned sensor chips by spin coating the 2% PS solution at 2000 rpm for 30 s and drying in vacuo for 24 h. The PS surface was oxidized by oxygen plasma treatment under 13 Pa oxygen with a power of 33 W for 10 s, and was left in the atmosphere overnight. On the surface of the oxidized PS-coated sensor chip, 5 μL of a 55% monomer solution containing only IPAAm or a mixture of IPAAm/CIPAAm (molar ratio 99:1) in 2-propanol was uniformly spread. Immediately, electron-beam irradiation using an area beam electron processing system (Curetron EBC-200-AA2; Nissin High Voltage, Kyoto, Japan) was carried out to promote the polymerization and covalent grafting of the polymer onto the PS surface [17,38]. After being immersed in water overnight at 4 °C, the surfaces were washed with high-pressure water to remove monomers and unimmobilized polymer, rinsed with water, and dried at 45 °C for 4 h. The used sensor chips after the measurements were washed with tetrahydrofuran to remove PS and cleaned, according to the manufacturer’s instructions. 

### 4.3. Heparin Immobilization on Poly(IPAAm-co-CIPAAm)-Grafted Sensor Chips

Heparin immobilization was carried out according to a previous paper [17]. Briefly, the surface of a poly(IPAAm-*co*-CIPAAm)-grafted sensor chip in a 35 mmφ dish was activated by 3 mM EDC/NHS in 10 mM MES for 24 h in a thermostatted shaker at 25 °C. Covalent immobilization of heparin onto the activated surfaces through ester formation was carried out in 50 µg/mL heparin in 10 mM MES for 24 h in a thermostatted shaker at 25 °C. The surfaces were thoroughly washed with water and were dried in air.

### 4.4. Surface Analyses

The PS-coated sensor chips were analyzed by spectral interferometry using a photonic multi-channel analyzer PMA-11 C7473-36 (Hamamatsu Photonics, Shizuoka, Japan) and a UV-Vis fiber light source L7893 (Hamamatsu Photonics). The optical constant and film thickness of PS were estimated from spectral interferometry with a film thickness measurement software U10339-01 (Hamamatsu Photonics). The PIPAAm and heparin-immobilized poly(IPAAm-*co*-CIPAAm)-grafted surfaces of the sensor chips were analyzed using X-ray photoelectron spectroscopy (XPS). High-resolution spectra of C_1s_, N_1s_, O_1s_, S_2p_, and Au_4f_ were collected at a take-off angle of 90° with an X-ray photoelectron spectrometer K-Alpha (Thermo Fisher Scientific), which was equipped with a monochromated Al Ka X-ray source (1486.6 eV) and a low-energy flood gun to neutralize the surface charge. The spot size of the monochromated X-ray was set to 400 μm in diameter. All of the peaks in the high-resolution spectra were referenced to a main C_1s_ peak at 285.0 eV. The elemental composition on the surfaces was calculated from the integral area of each peak with a Shirley background using Thermo Avantage ver. 4.88 software (Thermo Fisher Scientific).

### 4.5. Monitoring Temperature-Dependent Changes in Frequency and Dissipation Using the QCM-D System

Temperature-dependent frequency and dissipation shifts, Δ*f* and Δ*D*, on the prepared sensor chip surfaces were monitored using a QCM-D system (QSense Explorer E1-HO and QSoft 401 software, Biolin Scientific). A sensor chip was set in a flow module, and the inlet was connected with a peristatic pump and was equilibrated with PBS under a constant temperature and flow rate of 50 μL/min. Prior to QCM-D measurements, PBS was degassed by sonication under reduced pressure to prevent the formation of air bubbles in the flowing system. The temperature of the PBS reservoir was maintained at 40 °C. The flow module was heated and cooled between 20 and 40 °C at a rate of 0.1 °C/min.

### 4.6. Monitoring Serum Protein Adsorption Using the QCM-D System

Protein adsorption on the PIPAAm and Heparin-IC1 surfaces was monitored using a QCM-D system. While monitoring Δ*f* and Δ*D* at 20 or 37 °C, the solution of FN or BSA in PBS was added into the flow module at a rate of 50 μL/min for 60 min after the baseline shift was within 1 Hz/min for 10 min. Then, the surface in the flow module was rinsed with PBS for 15 min. FN and BSA solutions were prepared by the dilution of the stock solutions with PBS to obtain concentrations of 30 μg/mL and 4 mg/mL, respectively, which correspond to 10% of the physiological plasma concentrations [29]. The dilution was carried out in Sumilon Proteosave SS tubes (Sumitomo Bakelite, Tokyo, Japan) to reduce nonspecific adsorption. Prior to the QCM-D measurements, all of the solutions were degassed. For measurements at 37 °C, all of the solutions were heated to 40 °C. Oxygen plasma-treated PS (Oxidized PS) surfaces were used as a control.

To analyze the dynamic adhesion/de-adhesion of proteins, temperature-dependent Δ*f* and Δ*D* shifts were monitored on PIPAAm and Heparin-IC1 surfaces during stepwise temperature changes under a flow of 50 µL/min. Typically, after reaching a stable baseline within 1 Hz/min for 10 min at 20 °C, the temperature was changed to 37 °C for 30 min. The surfaces were contacted with BSA or FN solutions for 60 min at 37 °C and rinsed with PBS for 30 min. Finally, the temperature was decreased to 20 °C for 30 min. All of the solutions in the reservoir were thermostatted at 40 °C.

### 4.7. Estimation of The Thickness and Mass of The Adsorbed Serum Proteins

The thickness and mass of the adsorbed proteins were estimated using QTools software (Biolin Scientific). The time course of the thickness changes were obtained by curve-fitting using the Voight model [36], and the assumed parameters are as follows: fluid density, 1000 kg/m^3^; fluid viscosity, 0.001 kg/ms; adsorbed protein density, 1150 kg/m^3^. The fifth, seventh, and ninth overtones of Δ*f* and Δ*D* were used for the curve fitting. The adsorbed areal mass based on Voight modeling was estimated while using the following equation:

Δ*m*_Voight_ (ng/cm^2^) = 1150 (kg/m^3^) × thickness (m) × 10^8^ (1)

Additionally, the adsorbed areal mass was calculated based on the Sauerbrey equation, as follows:

Δ*m*_Sauerbrey_ (ng/cm^2^) = −17.7 × Δ*f*/*n* (2)

where *n* is the overtone number and Δ*f* is frequency shift at the *n*-th overtone between the baseline before the injection and the plateau after rinsing with PBS. Here, the fifth overtone of Δ*f* was used for the calculation. A value of Δ*m*_Sauerbrey_ below 17.7 ng/cm^2^ (i.e., Δ*f* < 1 Hz) is below the detection limit.

### 4.8. Statistical Analyses

All of the statistical analyses were conducted using RStudio software ver. 1.1.442 (RStudio Inc., Boston, MA, USA). Comparisons between two groups were carried out using the *t*-test. For the comparison of more than two groups, multiple comparisons between groups were made using the Tukey honestly significant difference (HSD) test. A *p*-value < 0.05 was considered to be statistically significant.

## 5. Conclusions

In this study, thermoresponsive surfaces were formed on PS-coated sensor chips to replicate thermoresponsive tissue-culture PS dishes. XPS measurements and temperature-dependent Δ*f* and Δ*D* values using QCM-D revealed that the thermoresponsive polymers were successfully grafted onto the oxidized PS thin films on the surface of the sensor chips. Temperature-dependent adsorption of BSA and FN on the thermoresponsive polymer-grafted surfaces was observed under static temperature. While the mass of adsorbed BSA and FN that was calculated using QCM-D was overestimated by a factor of 3–5, the values can be utilized for relative comparisons among the same substrate. More importantly, the thermoresponsive dynamic behavior of serum protein adsorption/desorption was monitored using QCM-D techniques. Observation of this dynamic behavior revealed that the adsorbed BSA and FN at 37 °C remained on both of the surfaces after decreasing the temperature to 20 °C. Further investigations are required to reveal the dynamic detachment process of the proteins in combination with cultured cells.

## Figures and Tables

**Table 1:** Elemental analysis of oxidized PS, PIPAAm, Heparin-IC1 surfaces on sensor chips ^1^.

Code	Atom (%) ^2^					N/C ^4^
	C	N	O	S	Au	
Oxidized PS	77.74 ± 0.40	1.83 ± 0.30	20.42 ± 0.33	0.01 ± 0.01	N.D. ^3^	0.02 ± 0.00
PIPAAm	80.15 ± 0.69	7.19 ± 0.97	12.63 ± 0.30	0.03 ± 0.02	N.D. ^3^	0.09 ± 0.01
Heparin-IC1	80.60 ± 0.55	6.95 ± 0.31	12.35 ± 0.28	0.09 ± 0.01	N.D. ^3^	0.09 ± 0.00

^1^ Take-off angle, 90°; ^2^ Values are expressed as the mean ± standard deviation (S.D.) of triplicate experiments; ^3^ N.D., not detected; ^4^ Theoretical N/C value for PIPAAm, 0.17.

**Table 2:** Thickness of adsorbed proteins on oxidized PS surfaces determined by using the Voigt model ^1^.

Code	Thickness (nm)	
	BSA	FN
Oxidized PS, 20 °C	4.5 ± 0.4	13.8 ± 1.0 **
Oxidized PS, 37 °C	4.2 ± 0.6	10.3 ± 0.1 **

^1^ Values are expressed as the mean ± S.D. of triplicate experiments; ** Statistically significant, *p* < 0.01.

## Abbreviations

QCM-D	Quartz crystal microbalance with dissipation
PIPAAm	Poly(*N*-isoprolylacrylamide)-grafted surface
Hparin-IC1	Heparin-immobilized poly(*N*-isoprolylacrylamide-*co*-2-carboxyisopropylacrylamide)-grafted surface
PS	Polystyrene
ECM	Extracellular matrix
FN	Fibronectin
BSA	Bovine serum albumin
XPS	X-ray photoelectron spectroscopy
